# HyperArc^TM^ Dosimetric Validation for Multiple Targets Using Ionization Chamber and RT-100 Polymer Gel

**DOI:** 10.3390/gels8080481

**Published:** 2022-07-31

**Authors:** Lucia Zirone, Elisa Bonanno, Giuseppina Rita Borzì, Nina Cavalli, Alessia D’Anna, Rosaria Galvagno, Andrea Girlando, Anna Maria Gueli, Martina Pace, Giuseppe Stella, Carmelo Marino

**Affiliations:** 1 Medical Physics Department, Humanitas Istituto Clinico Catanese, 95045 Catania, Italy; lucia.zirone@ccocatania.it (L.Z.); elisa.bonanno@ccocatania.it (E.B.); giuseppina.borzi@ccocatania.it (G.R.B.); nina.cavalli@ccocatania.it (N.C.); martina.pace@ccocatania.it (M.P.); carmelo.marino@ccocatania.it (C.M.); 2 Department of Physics and Astronomy E. Majorana, University of Catania, 95123 Catania, Italy; alessia.danna@phd.unict.it (A.D.); sara.galvagno@gmail.com (R.G.); anna.gueli@unict.it (A.M.G.); 3 Radiotherapy Department, Humanitas Istituto Clinico Catanese, 95045 Catania, Italy; andrea.girlando@ccocatania.it

**Keywords:** polymer gel, 3D-printed anthropomorphic phantom, HyperArc

## Abstract

Multiple brain metastases single-isocenter stereotactic radiosurgery (SRS) treatment is increasingly employed in radiotherapy department. Before its use in clinical routine, it is recommended to perform end-to-end tests. In this work, we report the results of five HyperArc^TM^ treatment plans obtained by both ionization chamber (IC) and polymer gel. The end-to-end tests were performed using a water equivalent Mobius Verification Phantom^TM^ (MVP) and a 3D-printed anthropomorphic head phantom PseudoPatient^®^ (PP) (RTsafe P.C., Athens, Greece); 2D and 3D dose distributions were evaluated on the PP phantom using polymer gel (RTsafe). Gels were read by 1.5T magnetic resonance imaging (MRI). Comparison between calculated and measured distributions was performed using gamma index passing rate evaluation by different criteria (5% 2 mm, 3% 2 mm, 5% 1 mm). Mean point dose differences of 1.01% [min −0.77%–max 2.89%] and 0.23% [min 0.01%–max 2.81%] were found in MVP and PP phantoms, respectively. For each target volume, the obtained results in terms of gamma index passing rate show an agreement >95% with 5% 2 mm and 3% 2 mm criteria for both 2D and 3D distributions. The obtained results confirmed that the use of a single isocenter for multiple lesions reduces the treatment time without compromising accuracy, even in the case of target volumes that are quite distant from the isocenter.

## 1. Introduction

Stereotactic radiotherapy (SRT) is a well-established and highly effective therapeutic modality for the treatment of brain metastases [1,2,3], benign brain tumors [4,5,6], and other neurological disorders [7]. The stereotactic technique is also suitable for the treatment of extracranial districts (stereotactic body radiation therapy–SBRT) such as lung, liver, and lymph nodes [8]. SRT is characterized by the delivery of high-dose radiation in a limited number of fractions or in a single session (stereotactic radiosurgery–SRS). SRS/SRT allows the obtaining of a highly conformal dose distribution to the target, minimizing the surrounding normal tissues toxicity through the use of non-coplanar arcs, small radiation fields, and non-homogeneous dose distributions [9,10]. Recently, interesting developments regarding SRS/SRT allowed the introduction of an innovative stereotactic technique, i.e., the HyperArc^TM^ (HA). HA is a non-coplanar volumetric modulated arc therapy (VMAT)–based technique employed for cranial SRT/SRS treatments; it uses a fixed geometry setup, which provides a high plan quality, especially for single-isocenter and multiple targets [11,12,13]. One of the advantages of care for multiple lesions with a single isocenter is the treatment time reduction, which also minimizes the likelihood of patient movement and noncompliance with sequential treatments for multiple fraction treatments [14]. Moreover, HA introduces the possibility of delivering treatment automatically, limiting user input as much as possible; during the treatment planning phase, even the positioning of the isocenter is performed automatically. In addition, a new normal tissue objective algorithm, the stereotactic radiosurgery normal tissue objective (SRS NTO), is set by default for HA plans during the optimization phase. SRS NTO controls dose fall-off and dose bridging between targets. It automatically recognizes targets spatial arrangements and tries to prevent dose bridging from occurring at least at dose levels higher than 17% of prescription [15]. 

Critical steps in starting a clinical program require due attention because of the complexity of SRS/SRT and of the HA technique. These include the introduction of quality assurance (QA) procedures which also include patient-specific QA (PSQA). The end-to-end test represents an ideal QA procedure for a new technique validation before its clinical implementation [16,17]. This test is based on treatment phases monitoring, i.e., the simulation, planning, delivery, and dose verification steps; during this test a phantom proceeds through the standard workflow like a real patient [16]. The goal is verifying the correct functioning of all treatment components in order to deliver the desired radiation dose in a more accurate way. Moreover, the use of small fields in stereotactic plans represents a crucial aspect for measurement execution in the end-to-end tests, due to several aspects that make small field dosimetry more difficult than the conventional one, such as loss of lateral charged particle equilibrium (LCPE), partial occlusion of radiation source, and volume averaging effect. In these situations, it is essential to use appropriate detectors with high spatial resolution, small size, and built with material suitable for minimizing the perturbation of the particle fluence. In addition, an ideal detector should have a linear response with dose, high stability, no directional and energy dependence, and should be tissue equivalent [18].

Ionization chambers are used in radiotherapy for point dose measurements, commissioning of the treatment unit, output calibration, and verification of the delivered dose thanks to their linear response, excellent stability, and independence of the dose rate and of the beam direction [19,20]. In stereotactic treatments, which employ high-dose gradients, inhomogeneous distributions, and very small fields, chamber size is an aspect not to be neglected; volumes that are too large tend to underestimate dose, and the effect is more pronounced as the active volume increases. Moreover, the chamber should be placed in a uniform dose region to minimize the effects of the average volume on a gradient region [21]. 

Polymer gels, instead, allow obtaining both 2D and 3D spatial information, compared to most other available dosimetry systems [22]. The interaction of several types of radiation with polymer gel dosimeters has been studied. The most studied types of irradiation are gamma rays from cobalt sources and high-energy x-rays produced by clinical linear accelerators. No significant energy dependence was found for photon beam energies between 6 MV and 25 MV for most of gel dosimeters [23]. These dosimeters are highly sensitive and do not present the problem of ion diffusion, typical of other gel dosimeters, such as Fricke gels [18]. However, their use in clinical routine is limited by problems in the production phase due to the high toxicity of the monomers required and their sensitivity to the presence of oxygen that inhibits the polymerization [24]. A polymer gel detector is obtained by mixing water with a gelling substance. A certain number of monomers and a crosslinking agent are then homogeneously dispersed within the resulting mixture. The characteristics of the gels and their response to radiation varies according to type and percentage of compounds incorporated [25]. Polymer gel dosimeters can be considered chemical dosimeters that rely on a radiation-induced chemical reaction. Upon irradiation, water molecules are dissociated into several highly reactive radicals and ions during a process termed ‘radiolysis’. These radiolytic products of water may react subsequently with the monomers inducing polymerization. [26,27,28,29,30,31]. The degree of polymerization is dose-dependent and can be assessed using nuclear magnetic resonance imaging (NMRI). The polymerization causes a reduction in the mobility of nearby water molecules; this affects typical NMR measurement parameters of water in the gel, in particular the spin–spin relaxation time (T2). Therefore, from the measurement of the MRI signal it is possible to determine the absorbed dose [32]. Although the polymer gels cannot be used as standard dosimeters because of reproducibility issues and advanced analysis techniques, some reports suggest that they are suitable for the measurements of output factors, beam profiles, and dose distributions in small fields due to their high spatial resolution. Yao et al. [33] investigated the dose distribution of flattering filter-free (FFF) and flattened beams for small field irradiation by using N-isopropylacrylamide (NIPAM) polymer gel, obtaining a gamma passing rate >90% for field sizes smaller than 2 × 2 cm^2^ with a 2% 2 mm criterion. Parwaie et al. [34] evaluated the efficacy of the first normoxic polymer gel, methacrylic and ascorbic acid in gelatin initiated by copper (MAGIC), in the measurement of dosimetric parameters beyond bone heterogeneity in small irradiation field. They also compared the obtained results with radiochromic films EBT3 and Monte Carlo simulations; regarding percentage depth dose (PDD), the best achievements are with gels. The composition of MAGIC was optimized by introducing formaldehyde, resulting in MAGIC-f which has better temporal stability and limits the problem of diffusion. The characteristics of MAGIC-f were studied by Azadeh et al. [35], who evaluated dose distributions, penumbra width, and small field output factors. Results show that these parameters are in good agreement with EBT3 films. Another type of polymer gel, the normoxic polyacrylamide gelatin (n-PAG), which is characterized by high dose sensitivity and spatial resolution, was investigated by Kudrelicius et al. [36]. The performed evaluations indicate a possible application of n-PAG as a QA tool in SRS treatments. There is therefore a wide variety of polymeric gels on the market, with chemical compositions that are continuously optimized to reduce these problems and improve their performance in the radiotherapy field. Polymer-gel dosimeters containing N-(Hydroxymethyl) acrylamide (NHMA) with different concentrations of potassium chloride (KCl) were developed and introduced for use in radiotherapy by Rabaeh et al. (2021a) [37]. The dosimeter was found stable within a period of 2–120 h after irradiation, and it is independent of dose rate in the range of 50–600 cGy/min and independent of photon beam energy between 6 and 15 MV within 7.5% overall uncertainty. Polymer gels that contain a N,N′-methylene-bis-acrylamide (BIS) crosslinker without the need of adding another radiosensitive monomer have been introduced as a new low-toxic polymer gel dosimeter [38]. The gel dosimetry accuracy was evaluated by calculating the overall uncertainty and found to be 7.04% (2σ, 95% confidence level). The effect of lithium chloride (LiCl) on the dose–response performance of the N-(3-methoxypropyl) acrylamide polymer-gel dosimeter (NMPAGAT) was studied for 3D dose measurements. Results show that the R2 dose–response of NMPAGAT–LiCl gels improved with increasing the concentration of LiCl [39].

The aim of this work, carried out at Humanitas–Istituto Clinico Catanese, is the dosimetric validation of the HyperArc^TM^ technique, through end-to-end tests on five multi-target single-isocenter SRS plans. For all plans, the tests were performed using an Ionization Chamber (IC) CC04 (IBA Dosimetry, Germany) and a polymeric gel (RTgel-100) to obtain point measurements and 2D and 3D distribution of the delivered dose. Measurements with the ionization chamber were performed on both simple geometry phantom and anthropomorphic phantom. The agreement between the calculated and measured dose was evaluated in terms of the average dose (D_mean_) over the sensitive volume of the chamber. The same anthropomorphic phantom was used for end-to-end testing with polymer gels. In this case, the comparison between the two distributions was performed through dose profiles and through 2D and 3D gamma index passing rate analysis, relative to the various lesions. 

## 2. Materials and Methods 

### 2.1. End-to-End Tests with Ionization Chamber

SRS treatment plans were calculated with Acuros 15.6.06 algorithm (calculation grid size = 1.25 mm) on Eclipse Varian Medical Systems TPS 15.6 and in VMAT mode; a 6 MV photon beam was used in FFF mode and maximum dose rate of 1400 Monitor Unit/minute (MU/min). The five plans evaluated include one plan with a single target and four plans with multiple targets (2–5). Plan parameters are shown in Table 1. A total of 4 non-coplanar fields were used for all plans with gantry angles ranging from 180.1° to 179.9° and couch rotation of 0°, ±45°, ±90°. 

The planning approach requires that the isodose curve corresponding to 90% of the prescription dose covers at least 99.5% of each planning target volume (PTV). For the M4 plan, the 90% isodose is relative to the maximum prescription dose (D_p_).

The ionization chamber used is a CC04 (IBA Dosimetry, Germany) with an active volume of 0.04 cm^3^. 

Measurements were performed on two phantoms: the water equivalent (Plastic Water^TM^–0,5%) Mobius Verification Phantom^TM^ (MVP) of 23 cm × 26 cm × 10 cm, equipped with 7 inserts (A, B, C, D, E, F, G) that allow the positioning in various points of different types of ionization chambers and a 3D-printed anthropomorphic phantom based on CT images of a generic patient and faithfully reproduces the patient’s anatomy named PseudoPatient^®^ (PP) and produced by RTsafe P.C. (Athens, Greece). The PP is filled with water and equipped with specific inserts that ensure the housing of various types of detectors, allowing us to perform point, 2D and 3D dose measurements.

CT images were acquired in axial mode for both phantoms, with the same slice thickness of 1.25 mm as set for the patient. To reproduce the same conditions of an SRS treatment, a thermoplastic mask, typically used for these treatments, was specifically modeled on the PP. Additionally, the PP phantom was placed on the encompassing structure.

**Figure 1:**
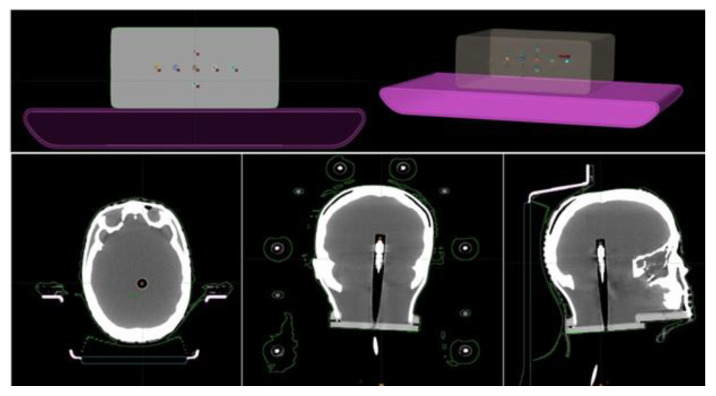
Axial and 3D plane visualization of the MVP phantom (**top**) and axial, coronal, and sagittal plane visualization of CT images of the PP phantom (**bottom**). In each phantom the structures corresponding to the positions of the ionization chamber were contoured.

For each treatment plan, a verification plan was generated and calculated on phantoms CT images. Structures corresponding to the insert in which the chamber is allocated were contoured on the CT images, with volume of 0.04 cm^3^ (Figure 1). The problem of using chambers with small volumes, such as the CC04, arises when there are dose gradients near the chamber. In this condition, even small positioning errors might cause large differences between the measured and the calculated dose. For this reason, planes were calculated so that the center of each PTV coincides with the center of the active chamber volume. A total of 15 verification planes were then calculated for each phantom. 

The calculated dose value is the D_mean_ of contoured structures. The measured dose was derived from the charge measured by the chamber, applying the appropriate correction factors in accordance with the formalism used in the report IAEA no. 398 [40]. 

Verification plans were delivered in QA mode through a Varian TrueBeam 2.7 linear accelerator with High-Definition MLC. Phantoms were then set up with Cone-Beam CT (CBCT) image guidance and irradiated according to the treatment plan.

### 2.2. End-to-End Tests with Polymer Gels

The polymer gel (RTgel-100) used for the end-to-end tests were produced by RTsafe P.C. (Athens, Greece). Further characterisation of the N-vinylpyrrolidone-based polymer gel (VIP gel) can be found in the literature [41].

On arrival, the gels are contained within the cylindrical (Poly Methyl MethAcrylate) PMMA insert specifically for phantom PP at a temperature of approximately 15°. Gels were then maintained at a temperature of between 20° and 24° during storage, irradiation, and imaging processes to ensure the correct gel consistency. The phantom used for the test is the same PseudoPatient^®^ described previously for the CC04 measurements; however, the insert related to the ionization chamber was replaced with the cylindrical vials containing the gel. 

**Figure 2:**
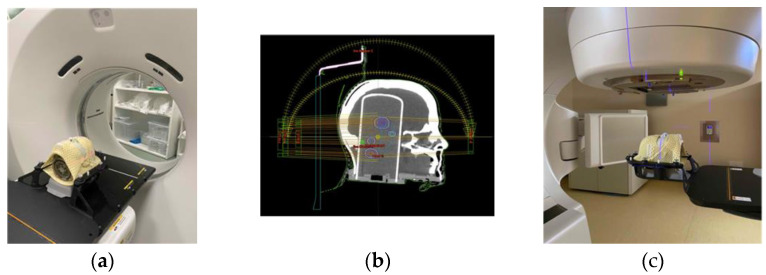
End-to-end workflow with polymeric gels: (**a**) acquisition of the CT images of the PP phantom; (**b**) creation of the verification plan; (**c**) delivery of the verification plan.

Once the correct temperature was reached, CT images of the PP phantom containing the cylinder with the gel placed on the encompassing system were acquired (Figure 2a); acquisition was obtained using a slice thickness of 1.25 mm in axial mode.

The next day, the verification plans were created and delivered (Figure 2b,c). SRS treatment plans, evaluated with the polymer gels, are the same as those already considered in dose measurements with the ionization chamber. In contrast to this, the prescription dose was reduced so that the maximum dose did not exceed 12 Gy to ensure dose-response linearity of dosimeter; general information about the plans is shown in Table 2. 

An MRI scan was acquired 24 h post irradiation using a Philips Achieva dStream 1,5T scanner (Philips Healthcare, Best, Netherlands). MR images of the PP phantom were acquired by implementing a 3D T2w multi-echo turbo spin echo (TSE) pulse sequence suggested by the gel company, using 20 echo times between 25 ms e 785 ms in steps of 40 ms and repetition time (TR) and flip angle (FA) of 2000 ms and 90°, respectively. The scan length was set to include the entire cylinder; as a result, the duration of each individual scan was approximately 40 min. 

T2 maps were derived from the acquired MR images using the “MR Analysis Calculator” plugin of ImageJ software (1.53 k). The plugin calculates T2 maps through a pixel-by-pixel evaluation of MRI images, considering individual echo times. Calculation is performed through an algorithm that fits the data of each slice according to Equation (1):

(1) $$T_{2}=\frac{-TE_{n}}{lnln\;\frac{S_{n}}{S_{0\;\left(T_{1},\rho \right)}}\;}$$

where *S_n_* is the signal intensity of each pixel corresponding to the n-echo time (*TE_n_*) and *S*_0_(*T*_1_, ρ) is the signal saturation factor (pseudodensity). The *T*_2_ maps were then manually registered to the planning CT (rigid registration; translation + rotation), using the geometrical bone-mimicking structures of the phantoms. Due to the inverse relationship between *T*_2_ and absorbed dose, the inverse of the *T*_2_ map (1/*T*_2_ = *R*_2_) is linearly dependent on the absorbed dose [42].

No polymer gel dose calibration was performed, and we relied instead on normalization of the polymer signal. Following the procedure outlined in Lukas Nierer et al., 2022 [43], the maximum R2 value of the PTV was normalized to the TPS PTV maximum dose. To account for the inherent baseline R2 signal of the polymer, gel which is distant from the beam paths received a very limited scatter dose. This low-dose signal can be defined as baseline signal in good approximation.

The qualitative assessment was made for each target by comparing calculated and measured dose profiles in the axial plane.

A common method for performing a quantitative comparison of calculated and measured distributions is the calculation of the Gamma Index Passing Rate (GIPR), first introduced by Low et al. [44,45]. The Gamma Index (GI) quantifies the difference between measured and calculated dose distributions on a point-by-point basis by combining two acceptability criteria: the dose difference (DD) and the distance to agreement (DTA). DD (%) and DTA (mm) represent the percent dose difference and minimum distance between two points of equal dose, respectively. The GIPR is defined as the percentage of points satisfying the condition GI < 1.

In this study the quantitative evaluation was performed by 2D and 3D gamma analyses; 2D GIPR was calculated within a circular region of interest (ROI) containing each target. Isodose curves relative to the calculated and measured dose distributions were derived for each volume. 

The MRI images were DICOM-transferred to RTsafe for postprocessing, which converted the *T_2_* maps of the 3D MRI scan of the PP phantom into 3D-dose distribution measurements and performed a 3D gamma analysis. Analysis was performed within a volume of interest including the target and a portion of adjacent tissue. For each target, the histograms of the 3D GIPR for the different criteria were also obtained.

In both 2D and 3D analyses, the criteria were chosen as follows: 3% 2 mm [46], 5% 2 mm, and 5% 1 mm [46]. 

## 3. Results

### 3.1. Ionization Chamber Measurements 

Table 3 and Table 4 show the results obtained by end-to-end tests with CC04 ion chamber on MVP and PP phantom, respectively. For each target, the tables report the dose calculated by the TPS measured dose and dose difference (%) between these. The measured dose value was corrected considering the machine daily output. 

The dose difference mean value (%) is equal to 1.01% (max −2.89% and min −0.07%) for measurements performed on MVP and 0.23% (max 2.81% and min 0.01%) for measurements on the PP phantom. 

### 3.2. Polymer Gel Measurements

**Figure 3:**
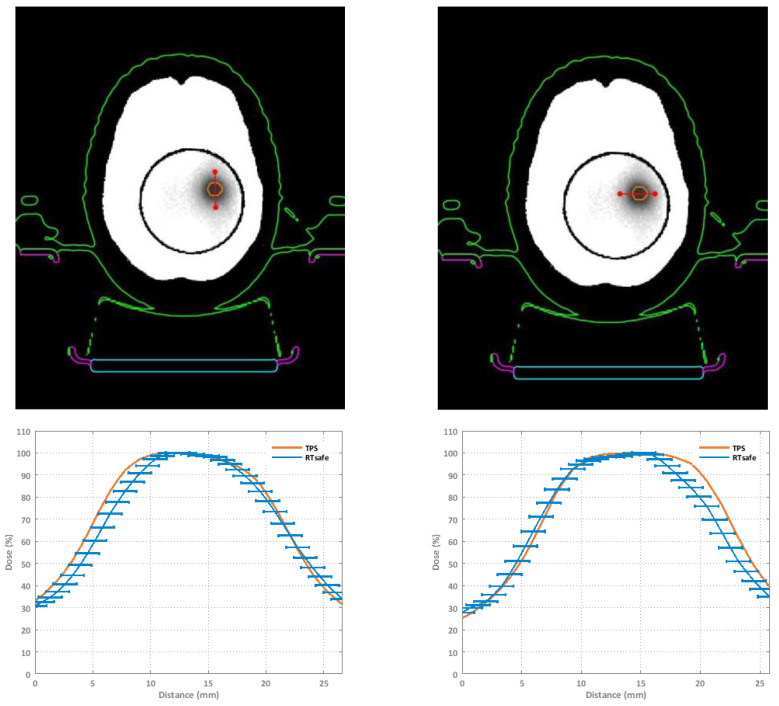
Orthogonal dose profiles comparison of a target that is 6.8 cm away from the isocenter. High-dose regions correspond to darker areas. Error bars correspond to ±1 mm spatial uncertainty.

**Figure 4:**
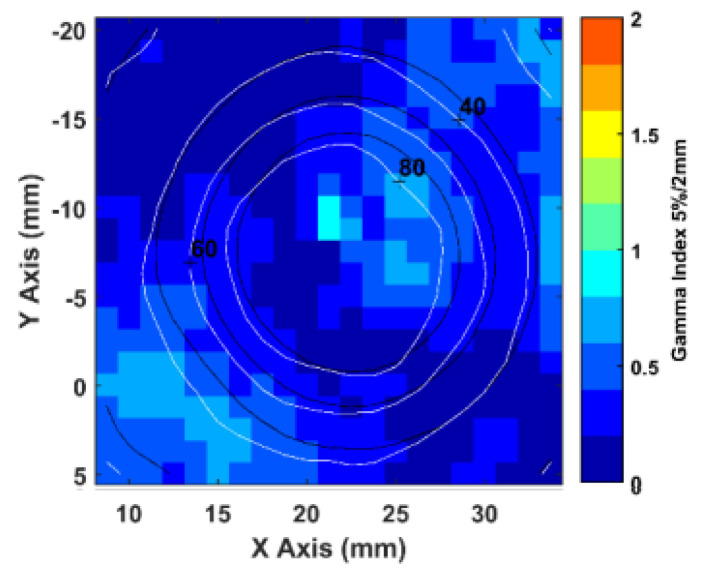
Example of gamma index map (5% 2 mm criteria) and the superposition of the calculated (dark blue lines) and measured (light blue lines) isodoses of the R2 maps, for a square ROI of an axial image encompassing the same target.

**Figure 5:**
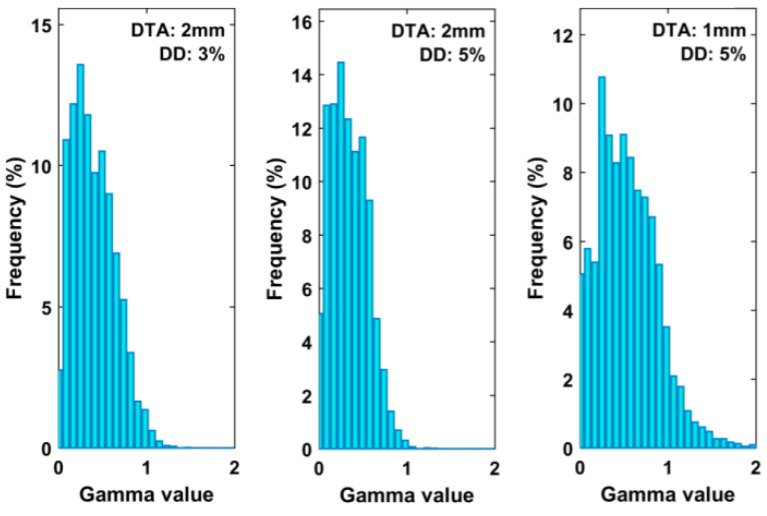
Histograms for the calculated gamma values of the 3D GI comparison test using the different passing criteria.

The comparison between calculated and measured dose distributions were made in terms of GIPR. In this case, the DD relative to the maximum dose for all pixels was calculated. Figure 3 shows an orthogonal dose profile comparison of the furthest target from the isocenter. This target is relative to the M3 plan and is 6.8 cm away from the isocenter. Figure 4 shows an example of a gamma index map (5% 2 mm criteria) and the superposition of the calculated (dark blue lines) and measured (light blue lines) isodoses of the R2 maps, for a square ROI of an axial image encompassing the same target. Figure 5 shows the 3D GI histograms for the different passing criteria. 

For uncertainty budget evaluation, the work of Awad et al. (2019) [47] was followed. Spatial registration uncertainties were set to 0.5 mm due to half the lateral resolution values. A temperature variation uncertainty of 1 °C was used to account for potential temperature drifts between pre- and post-irradiation between the CT and MR setup. The reproducibility of R2 was determined as a typical standard deviation of measured OD intensity within a region of uniform dose. 

Table 5 and Table 6 show the mean GIPR values, the standard deviation, and the minimum and the maximum values obtained for 2D and 3D gamma analysis for different DTA and ΔD criteria. 

In the case of the 2D analysis, the mean passing rate is higher than 95% for both 3% 2 mm and 5% 2 mm criteria, while for 5% 1 mm, it is below 90%. The 3D gamma analysis confirms the results of the 2D analysis, but for the 5% 1 mm criterion the calculated gamma passing rate is greater than 90%.

## 4. Discussion

The present study reports the measurements carried out for the dosimetric validation of the HyperArc^TM^ technique to introduce it in our clinical routine. For this purpose, end-to-end tests were performed to assess the entire treatment chain (CT simulation, treatment planning, treatment delivery, and dose verification) [48]. 

Measurements were performed with an ionization chamber suitable for small fields, to achieve point dose and a polymer gel to obtain 2D and 3D dose distributions. The point dose was measured both in a phantom with a simple geometry and an anthropomorphic phantom. The 2D and 3D dose distributions were obtained by inserting the polymer gel inside the anthropomorphic phantom. Particular attention was paid to SRS treatments with single isocenter and multiple lesions.

The results of the end-to-end ionization chamber tests, expressed in terms of average dose in the detector-sensitive volume, show a good agreement between measured and calculated doses. The deviations between calculated and measured doses were always below 3%, for both phantoms, in accordance with the recommendations of the AAPM TG No. 218 [46]. 

The absolute dose delivery accuracy suggests a clinically acceptable degree of dosimetric precision with the HyperArc technique for an end-to-end test. 

Polymer gels are high resolution dosimeters that allow us to obtain both 2D and 3D dose distributions. Additionally, polymer gels allow us to solve some problems related to small field dosimetry. Their main advantage comes from their tissue equivalence and the possibility of modeling them according to phantom shape. These characteristics reduce both the problems of fluence perturbation and detector positioning. 

Planar and volumetric dose distributions were assessed by 2D and 3D GIPR analysis using various criteria. 

According to obtained results, the average gamma passing rate is greater than 97% for both 2D and 3D cases. The average GIPR for 5%2mm is over 98% in both 2D and 3D cases, with a difference of 0.89% between planar and volumetric distributions. Finally, the 5% 1 mm evaluation resulted in an average GIPR of less than 95% for both 2D and 3D distributions.

Moreover, the obtained results agree with other works reported in the literature related to mono-isocentric SRS brain treatments with multiple targets. Saenz et al. [49] evaluated the accuracy of this type of treatment by end-to-end testing with an ionization chamber and polymer gels among various institutions. The max point difference was 1.7% and the 3D gamma passing rate values were greater than 90% with the 3% 2 mm criterion. In another study by Chang et al. [50], the difference obtained with the ionization chamber are about 3% and the 3D gamma passing rate values are greater than 99% with the 3% 3 mm criterion. The 3D gamma passing rate values are also comparable with those obtained by Bry et al. [51], who evaluated the accuracy of the IGRT positioning system in the case of a five-target treatment.

## 5. Conclusions

The end-to-end tests allowed us to assess the different components of the treatment chain. The information obtained from both the ionization chamber and polymer gel measurements confirmed that the use of a single isocenter for multiple lesions reduces the treatment time without compromising accuracy, even in the case of target volumes that are quite distant from the isocenter. According to the obtained results, the dosimetric validation enables the introduction of the HyperArc^TM^ technique into our clinical routine.

## Figures and Tables

**Table 1:** Characteristics of SRS plans evaluated with CC04 ion chamber.

ID	N° Target	V_target_ [cm^3^]	D_p_ [Gy]	Distance from ISO [mm]
S1	1	3.37	20	-
M1	2	0.47–0.93	20	21.8–22.9
M2	3	0.29–2.32	20	3.3–19.9
M3	4	1.49–1.57	21	28.00–68.21
M4	5	0.15–3.94	15–22	15.93–28.29

**Table 2:** Characteristics of SRS plans evaluated with polymer gel.

ID	N° Target	V_target_ [cm^3^]	D_p_ [Gy]
S1	1	3.37	7
M1	2	0.47–0.93	8
M2	3	0.29–2.32	9
M3	4	1.49–1.57	9
M4	5	0.15–3.94	6–9

**Table 3:** Dose values calculated by the TPS, measured with the CC04 chamber in the MVP, and respective percentage differences.

ID	Target	DTPS (Gy)	DMVP (Gy)	Diff. (%)
S1	PTV1	23.871	24.498	2.63
M1	PTV1	21.701	22.153	2.08
	PTV2	24.205	23.584	−2.57
M2	PTV1	23.755	24.008	1.07
	PTV2	24.129	23.944	−0.77
	PTV3	24.419	23.844	−2.36
M3	PTV1	25.491	24.721	−2.13
	PTV2	27.471	26.779	−2.52
	PTV3	26.845	26.595	−0.93
	PTV4	27.871	27.425	−1.60
M4	PTV1	26.183	25.397	−2.28
	PTV2	28.891	27.865	−2.74
	PTV3	20.257	19.671	−2.89
	PTV4	27.327	27.750	1.55
	PTV5	29.793	29.322	−1.58

**Table 4:** Dose values calculated by the TPS, measured with the CC04 chamber in the PP phantom, and respective percentage differences.

ID	Target	DTPS (Gy)	DPP (Gy)	Diff. (%)
S1	PTV1	23.297	24.067	2.81
M1	PTV1	21.847	21.957	0.51
	PTV2	22.261	22.426	0.74
M2	PTV1	23.654	24.269	2.60
	PTV2	22.672	22.769	0.43
	PTV3	22.429	22.623	0.87
M3	PTV1	24.808	24.264	−2.19
	PTV2	26.101	25.738	−1.39
	PTV3	25.837	25.529	−1.19
	PTV4	26.778	26.408	−1.38
M4	PTV1	23.972	24.243	1.13
	PTV2	26.741	26.647	−0.35
	PTV3	19.009	19.007	0.01
	PTV4	26.577	26.821	0.92
	PTV5	28.495	28.478	−0.06

**Table 5:** Gamma passing rate mean value (Mean), standard deviation (SD), minimum (Min) and maximum (Max) values for each passing criteria obtained by 2D gamma analysis.

Passing Criteria	2D Gamma Passing Rate			
	Mean (%)	SD (%)	Min (%)	Max (%)
3% 2 mm	97.96	1.09	96.39	100.00
5% 2 mm	99.78	0.55	97.89	100.00
5% 1 mm	84.15	13.32	60.53	98.39

**Table 6:** Gamma passing rate mean value (Mean), standard deviation (SD), minimum (Min) and maximum (Max) values for each passing criteria obtained by 3D gamma analysis.

Passing Criteria	3D Gamma Passing Rate			
	Mean (%)	SD (%)	Min (%)	Max (%)
3% 2 mm	97.92	2.28	92.76	100.00
5% 2 mm	98.89	1.66	94.25	100.00
5% 1 mm	91.38	10.24	73.28	100.00
